# Functional Strength in Children with Developmental Disabilities: An Exploratory Comparison of Three Clinical Cohorts Including Children with Developmental Coordination Disorder, Intellectual Disabilities, and Cerebral Palsy

**DOI:** 10.3390/sports14100465

**Published:** 2026-10-20

**Authors:** Wendy Aertssen, Bert Steenbergen, Bouwien Smits-Engelsman

**Affiliations:** 1 Master Specialized Physical Therapy, Habeo+ University for Professionals, 4818CP Breda, The Netherlands; 2 Behavioural Science Institute, Radboud University, 6525XZ Nijmegen, The Netherlands; b.steenbergen@pwo.ru.nl; 3 Department of Natural Sciences in Kinanthropology, Faculty of Physical Culture, Palacký University, CZ-77900 Olomouc, Czech Republic; bouwiensmits@hotmail.com; 4 Physical Activity, Sport and Recreation, Faculty Health Sciences, North-West University, Potchefstroom 2520, South Africa

**Keywords:** functional strength, FSM, task-specific, developmental coordination disorder, intellectual disabilities, cerebral palsy

## Abstract

**Background**: This study presents an exploratory comparison of data from three previously collected clinical cohorts. The first aim was to compare functional strength performance among a cohort of children with Developmental Coordination Disorder (DCD), Intellectual Disabilities (ID), and Cerebral Palsy (CP). The second aim was to examine whether the associations between age and functional strength performance differed across these cohorts. **Methods**: Data from 103 children aged 7–10 years (29 DCD, 47 ID, 27 CP) were combined. Functional Strength Measurement (FSM, FSM-ID, FSM-CP) scores were compared with reference norms. Cohort differences were examined using multivariate analysis of covariance (MANCOVA) with body weight included as a covariate. Associations between age and FSM performance were examined using General Linear Models including age, clinical cohort, and age-by-cohort interaction terms. **Results**: Compared with the FSM reference norms, 17–89% of participants were classified as impaired or at risk for muscle-power performance, while 62–92% demonstrated limitations in muscle-endurance tasks. Significant differences between the three clinical cohorts were observed for overarm throwing, underarm throwing, lifting a box, and stair climbing, with medium effect sizes. Evidence for age-by-cohort interactions was observed for overarm throwing, underarm throwing, standing long jump, and sit-to-stand performance, indicating that associations between age and functional-strength performance differed across clinical cohorts. **Conclusions**: Children in the clinical cohorts demonstrated lower levels of functional-strength performance than typically developing peers. Functional-strength profiles differed across clinical cohorts. However, as the data were derived from three distinct cohorts rather than collected within a single study, and minor differences exist between the FSM, FSM-ID and FSM-CP, the findings should be interpreted with caution and considered hypothesis-generating rather than confirmatory. Furthermore, because the data were cross-sectional, no conclusions can be drawn regarding developmental changes over time. These findings may help generate hypotheses for future assessment, intervention and longitudinal research in children with developmental disabilities.

## 1. Introduction

Good physical fitness is essential for all children, both with and without disabilities, as it plays a critical role in promoting participation in daily activities and sports, while also reducing the risk of secondary health problems such as obesity and cardiovascular disease [1,2]. For children with motor impairments, the ability to engage in active play and sports is often limited, leading to lower self-efficacy [3,4], increased sedentary behavior [5,6] and decreased levels of health-related physical fitness compared to typically developing (TD) children [7,8]. Identifying children with disabilities who have low fitness levels and enrolling them in appropriate programs may help improve their physical fitness.

Active play and sports activities for children are typically characterized by short bursts of high-intensity activity interspersed with periods of lower intensity [9]. Key components of muscular fitness—strength, muscle power, and muscle endurance—are crucial for performing everyday physical activities, such as jumping, pushing a swing, or throwing a ball [9]. Muscular strength refers to a muscle’s ability to generate maximal contraction, while muscle power describes the amount of work a muscle can perform over time, or the product of force exerted and the action velocity. Muscle endurance refers to the ability to sustain or repeat muscle contractions over a specific period. Recent guidelines increasingly recognize the importance of these muscular fitness components in promoting youth health, suggesting that muscular fitness should receive greater emphasis within youth activity recommendations [10].

Muscular fitness can be assessed in various ways. The International Classification of Functioning, Disability and Health (ICF) framework provides a conceptual approach to understanding how diseases and disabilities affect functioning [11]. Within this framework, impairments are described at different levels: body function and structure, activity, and participation. Each of these levels requires different measurement tools. Research by Ferguson et al. (2014), for example, demonstrated significant differences in the functional strength between children with Developmental Coordination Disorder (DCD) and TD peers when assessed at the level of activities, whereas no differences were found when assessed at the level of body function using isometric strength tests [12]. The strength of an individual muscle group does not provide direct information about its functional use in real-life situations. These findings suggest that functional assessments may provide more ecologically valid measures of muscular fitness, as they reflect the child’s ability to perform everyday tasks that require strength. Nevertheless, a limitation at the level of body function (strength, ROM, spasticity, muscle tone, coordination, or cognitive impairment) may influence activity outcomes. An activity involves the use of multiple muscle groups working together in a coordinated manner across a range of joint angles over a certain time period. Functional activities therefore provide an integrated representation of performance in which multiple underlying factors may contribute.

Children with DCD, intellectual disabilities (ID), and cerebral palsy (CP) often exhibit lower levels of muscular fitness than their TD peers [13,14,15,16]. However, the specific constraints—whether neurological, intellectual, or related to motor coordination—that influence their performance in a task requiring high levels of functional strength may differ. Comparing functional-strength profiles across these cohorts may therefore provide clinically relevant information regarding the activities that appear particularly challenging for each population. Such comparisons may also generate hypotheses regarding factors that contribute to functional-strength limitations and support the development of more tailored intervention approaches.

This study presents an exploratory comparison of data from three previous studies [17,18,19] that examined the psychometric properties of the Functional Strength Measure (FSM) in separate clinical populations. The FSM includes tasks that assess muscle power (e.g., standing long jump, overarm throwing) and muscle endurance (e.g., stair climbing, lifting a box), and has been shown to be a valid tool for measuring functional strength in both TD children and those with DCD, ID, or CP [17,18,19,20]. Previous intervention studies also indicate that the FSM is sensitive to change in these populations [21]. Despite these findings, direct comparisons between these clinical cohorts have not yet been undertaken, limiting our understanding of whether distinct functional-strength profiles emerge across developmental disabilities. Such comparisons may help clinicians identify activities that represent particular challenges for specific populations and may generate hypotheses regarding factors that contribute to differences in functional-strength performance. Understanding these patterns may assist the development of more targeted assessment and intervention strategies.

The first aim was to explore differences in functional-strength performance across three previously collected clinical cohorts of children with DCD, ID and CP.

Based on the previous literature, we expected the CP cohort to demonstrate lower functional-strength performance than the other cohorts [15,16]. In contrast, children in the DCD cohort are expected to show less pronounced deficits, as their main challenge lies in acquiring motor skills rather than muscle strength. Children included in the study with mild intellectual disabilities may not show obvious musculoskeletal problems, although low muscle tone is commonly observed [22,23]. Furthermore, previous research has shown that the relative loss of fitness due to sedentary behavior tends to be more pronounced in older children with these conditions [15,17,24,25]. It remains unclear, however, whether this trend also applies to functional muscle strength. Therefore, the second aim of this study was to examine whether associations between age and FSM scores differed across the three clinical cohorts.

## 2. Materials and Methods

### 2.1. Participants

This retrospective study combined data from three previously published studies [17,18,19], each focusing on a distinct clinical population. Data from children aged 7–10 years were included. Children were included in the DCD study if they met all criteria for DCD as outlined in the DSM-5. For children in the ID study, the primary inclusion criterion was mild intellectual disability (IQ between 50 and 70). Children were included in the CP study if they had spastic, dyskinetic, or ataxic CP, either unilateral or bilateral, and were classified at Gross Motor Function Classification System (GMFCS) levels I–II, Manual Ability Classification System (MACS) levels I–III, with an IQ above 70. The children were recruited from special needs schools, private pediatric physical therapy practices and rehabilitation centers.

A total of 103 participants were included: 29 children with Developmental Coordination Disorder (DCD; mean age 8.48, SD 1.06), 47 children with Intellectual Disabilities (ID; mean age 8.74, SD 1.24; mean IQ 58; 7 children with Down Syndrome, 4 children with other syndromes and 36 children without a specific syndrome), and 27 children with Cerebral Palsy (CP; mean age 8.44, SD 1.25; 20 children with GMFCS 1 of which 9 unilateral and 11 bilateral, 7 children with GMFCS II of which 1 unilateral and 6 bilateral. Of the 27 children with CP, 12 were classified at MACS level I, 13 at MACS level II and 2 at MACS level III.

The three studies had a cross-sectional design and were approved by the medical ethics committee (ECSW2014-3107-232). Informed consent was obtained from the parents of all children. Data collection took place from 2015 to 2018.

Demographic characteristics of the participants in each clinical cohorts are presented in Table 1.

### 2.2. Outcome Measure: Functional Strength Measurement (FSM)

The Functional Strength Measurement (FSM) is a norm-referenced tool designed to assess muscular fitness, including both muscle power and muscle endurance. The FSM consists of eight items, with four assessing muscle power (overarm throwing, standing long jump, underarm throwing, and chest pass) and four assessing muscle endurance (lateral step-up left and right, sit-to-stand, lifting a box, and stair climbing). Muscle power is measured by the distance (in cm) achieved on each task, while muscle endurance is measured by the number of repetitions completed within 30 s. Raw FSM scores can be converted to age-specific standard scores using reference values derived from a normative sample of more than 600 typically developing children aged 4–10 years. The standard scores are categorized as follows: 1 = impaired (≤5th percentile), 2 = at risk (6th–15th percentile), 3 = average low (16th–50st percentile), 4 = average (51st–85th percentile), 5 = above average (86th–95th percentile), and 6 = well above average (>96th percentile).

Some adaptations were introduced in the FSM-ID [18] and FSM-CP [19] to improve task comprehension and feasibility, these modifications were generally intended to reduce barriers unrelated to functional strength. For example, visual supports were added in the FSM-ID to facilitate understanding of task instructions, while children with CP were permitted to use compensatory strategies commonly employed in daily life. In the DCD study, the original FSM was used without adaptations.

The psychometric properties of the FSM, FSM-ID, and FSM-CP have been previously validated, demonstrating good reliability and validity across these populations [18,19,20]. A summary of the specific tasks for each version of the FSM is presented in Table 2.

The chest-pass item was omitted from the FSM-CP and could therefore not be included in the present comparison. Because children with CP typically have a more- and less-affected side, the analysis of the lateral step-up task was based on the better-performing and other side rather than on the left and right sides.

### 2.3. Procedure

The three studies followed the same FSM testing procedures. Children were assessed by different trained pediatric physical therapists across the three cohorts. All assessors were trained by the same instructor (WA) and used the same test manuals, standardized protocols and assessment materials. Testing was conducted in familiar environments (school, rehabilitation setting or pediatric physical therapy practice) in which the children routinely received educational or therapeutic services. Although the use of standardized procedures and identical assessment materials was intended to maximize comparability across the three cohorts, potential study-level influences cannot be fully excluded.

### 2.4. Statistical Analyses

Data were initially assessed for normality using the Shapiro–Wilk test. Age and body weight were not normally distributed across the three clinical cohorts, whereas the FSM item scores were normally distributed. Kruskal–Wallis tests revealed a significant difference in body weight among the three cohorts. Body weight was therefore included as covariate in the MANCOVA because it may influence performance on the functional strength items. Sex distribution did not differ significantly between cohorts and was not included as a covariate because the FSM assesses functional task performance without sex-specific scoring or differentiation. Age was examined separately as a primary variable of interest in the exploratory age-by-cohort analyses rather than being controlled for in the cohort comparison model.

Descriptive statistics were used to summarize the FSM scores for each group. The following analyses were conducted:

1. Comparison with Norms: The FSM scores for each clinical cohort were compared to the reference norms. These comparisons were visualized using radar charts to illustrate the differences between cohorts across the various FSM items.
2. Cohort Comparisons: A multivariate analysis of covariance (MANCOVA) was performed to examine differences in motor performance across the three clinical cohorts (DCD, ID and CP), with body weight included as a covariate. Prior to the MANCOVA, the relevant statistical assumptions were assessed including multivariate normality, linearity, homogeneity of regression slopes, and homogeneity of covariance matrices. Significant multivariate effects were followed by univariate analyses of covariance and Bonferroni-adjusted pairwise comparisons. Effect sizes were calculated using partial eta squared (ηp^2^), with the following interpretations: small ηp^2^ = 0.01, medium ηp^2^ = 0.06, and large ηp^2^ > 0.14 [26].
3. Age-by-cohort analyses: To examine whether the association between age and the different FSM-items differed between the three clinical cohorts, separate General Linear Models were conducted for each of the eight motor performance outcomes (raw scores). Age, cohort, and age-by-cohort interaction were included as predictors. The age-by-cohort interaction was used to assess whether the slope of the association between age and motor performance differed between the DCD, ID and CP cohorts. Regression coefficients (B), effect sizes (ηp^2^) and *p*-values were reported. The assumptions of linear regression, including linearity, homoscedasticity, normality of residuals, and the absence of influential observations, were assessed. Regression plots were created to visualize the association between the FSM and age. Because the data were cross-sectional, these slopes represent associations between age and test performance at a given point in time and should not be interpreted as developmental changes. Given the exploratory nature of these analyses and the number of interaction tests performed, the results of the age-by-cohort models should be interpreted with caution. No additional correction for multiple testing was applied across the eight age-by-cohort interaction models; therefore, these results should be considered hypothesis-generating rather than confirmatory.

The total study sample comprised 103 participants. However, the sample size varied across analyses because some children were unable to complete specific items, resulting in missing values in SPSS. For the MANCOVA, only participants with complete data for all variables included in the analysis were included. For the age-by-cohort analyses, participants with missing scores for a specific item were excluded only from the analysis of that item but remained eligible for analyses of other items for which valid scores were available. All statistical analyses were conducted using SPSS version 31, with a significance level set at α = 0.05.

## 3. Results

### 3.1. Scores of the FSM Compared to the Norms

**Figure 1:**
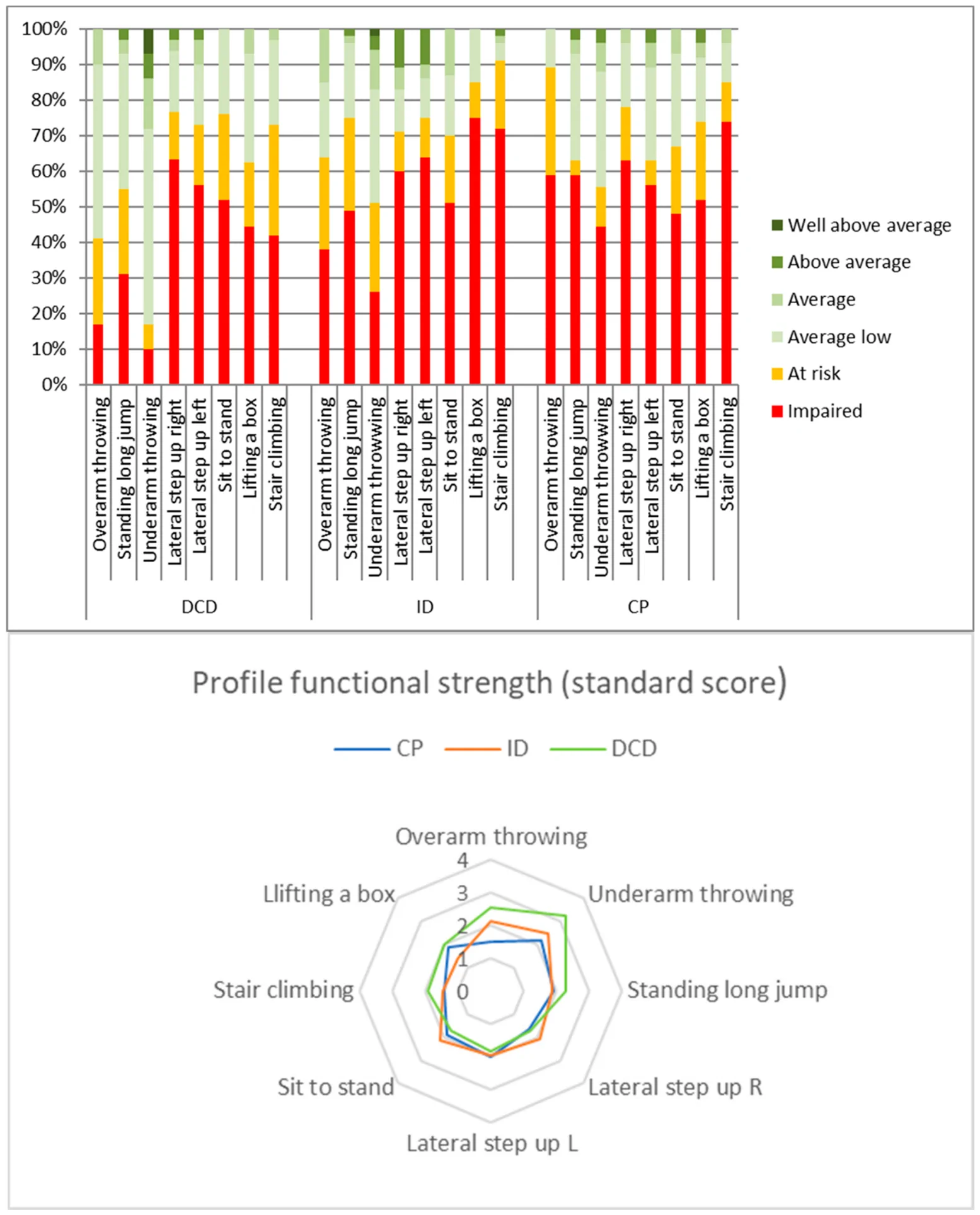
Functional-strength profiles of the three clinical cohorts relative to FSM reference values. Interpretation of the standard scores FSM: 1 = impaired, 2 = at risk, 3 = average low, 4 = average, 5 = above average, 6 = well above average.

For the muscle power items, 17–56% (DCD), 51–75% (ID), and 56–89% (CP) of the children scored “impaired” or “at risk” based on the reference norms (below the 16th percentile). For the muscle endurance items, the percentages were 62–76% (DCD), 70–92% (ID), and 67–85% (CP). Figure 1 shows the profiles of the different cohorts on the FSM items.

### 3.2. Differences Between the Clinical Cohorts

A multivariate analysis of covariance (MANCOVA) was conducted to examine differences between children in the DCD, ID and CP cohort across the eight outcomes of the FSM, with weight included as a covariate. The assumptions of multivariate normality, linearity, homogeneity of variance–covariance matrices, and homogeneity of regression slopes were assessed and considered to be adequately met. A significant multivariate effect of cohort was observed, Pillai’s Trace = 0.434, F(16, 162) = 2.81, *p* < 0.001, ηp^2^ = 0.217.

Follow-up univariate analyses revealed significant cohort effects for overarm throwing (*p* = 0.023), underarm throwing (*p* = 0.042), lifting a box (*p* = 0.015), and stair climbing (*p* = 0.010) with medium effect sizes (ηp^2^ = 0.070–0.100). No significant cohort effects were observed for standing long jump, lateral step-up best, lateral step-up other or sit-to-stand (all *p* ≥ 0.082).

Bonferroni-adjusted pairwise comparisons indicated significantly lower scores in the CP cohort than in the ID cohort for overarm throwing (*p* = 0.034) and underarm throwing (*p* = 0.047). For lifting a box, the score was significantly lower in the ID cohort than in the DCD cohort (*p* = 0.017). For stair climbing, the CP cohort had a significantly lower score than the DCD cohort (*p* = 0.008). No other pairwise comparisons reached statistical significance after Bonferroni adjustment. Table 3 presents the differences and effect sizes between the cohorts.

### 3.3. Functional Strength and Age

To examine whether the association between age and test performance (raw scores) differed between the three clinical cohorts (DCD, ID, CP), separate General Linear Models (GLM) were fitted for each test item, including age (in months), cohort, and age-by-cohort interaction. The estimated age-related slopes within each cohort and the omnibus tests of the age-by-cohort interaction are presented in Table 4. Significant age-by-cohort interactions were observed for overarm throwing (*p* = 0.046), standing long jump (*p* = 0.023), underarm throwing (*p* = 0.025), and sit-to-stand (*p* = 0.004) with medium effect sizes (ηp^2^ = 0.062–0.114). For lateral step-up other leg, the age-by-cohort interaction was at the conventional significance threshold (*p* = 0.050). No statistically significant age-by-cohort interaction was observed for lateral step-up best leg, lifting a box, or stair climbing. For the outcomes showing significant age-by-cohort interactions, estimated age slopes differed across clinical cohorts. For overarm throwing, estimated slopes were positive for CP (B = 0.782), ID (B = 2.732), and DCD (B = 1.481). For standing long jump, the estimated slope was negative for CP (B = −0.494) and positive for ID (B = 0.760) and DCD (B = 0.449). For underarm throwing, slopes were positive in all cohorts (CP: B = 0.744; ID: B = 3.858; DCD: B = 1.917). For sit-to-stand, the estimated slope was negative for CP (B = −0.139) and positive for ID (B = 0.107) and DCD (B = 0.075). Significant main effects of age were observed for overarm throwing, underarm throwing, lateral step-up best, lifting a box and stair climbing (all *p* ≤ 0.040). Table 4 presents the interaction of age, cohort and age-by-cohort on the different FSM-items. Because the data were cross-sectional, these slopes represent associations between age and test performance at a given point in time and should not be interpreted as longitudinal developmental changes (Table 4).

**Figure 2:**
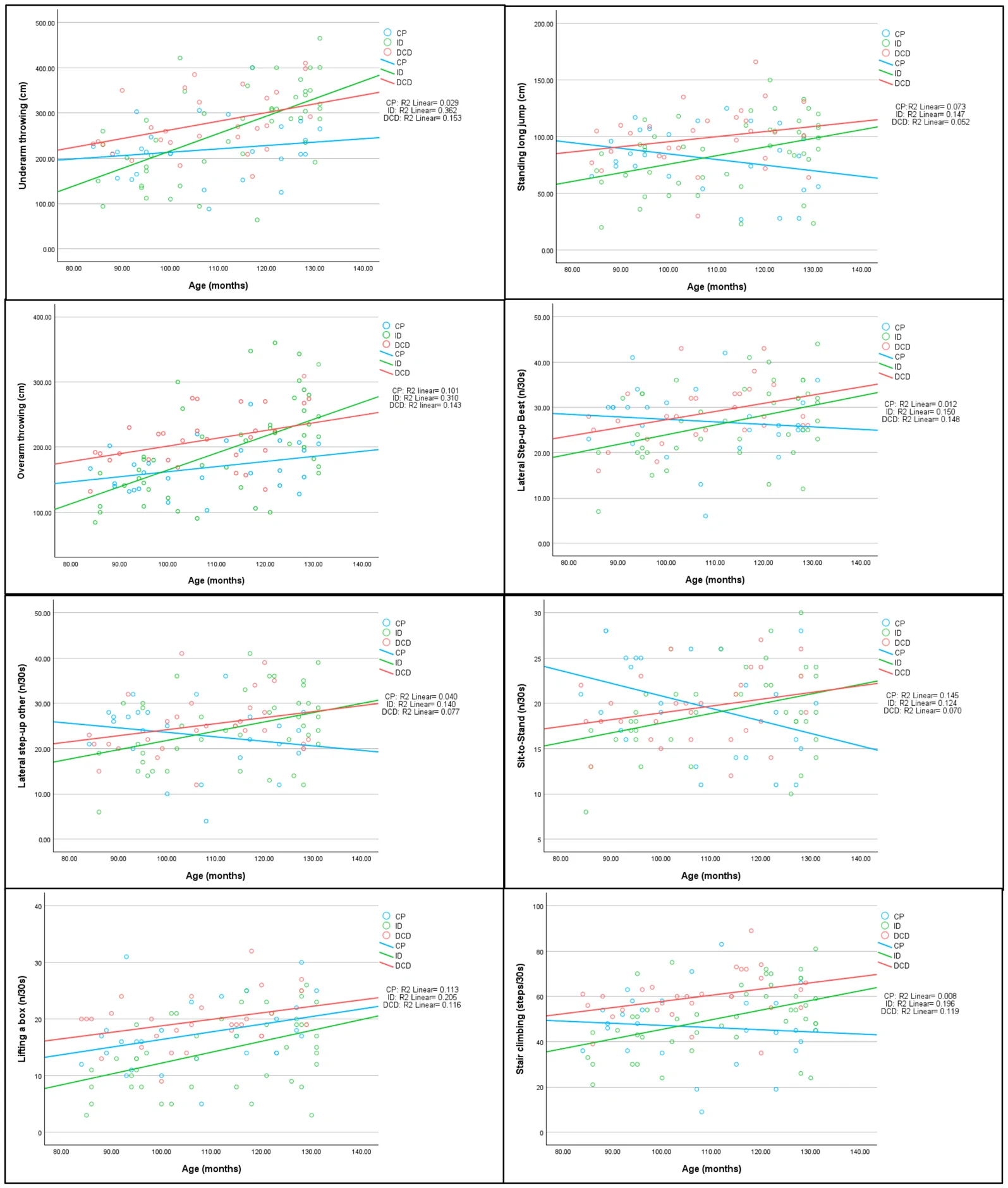
Scatterplots and regression lines of different items of the FSM in three clinical cohorts. CP = Cerebral palsy, ID = Intellectual Disabilities, DCD = Developmental Coordination Disorder. In the items standing long jump, lateral step-up, sit to stand and stair climbing the regression line in children with CP goes down.

To visualize the association between age and the raw test scores across the three clinical cohorts, scatter plots with cohort-specific linear regression lines were created (Figure 2).

## 4. Discussion

### 4.1. Overview of Findings

In this exploratory retrospective study, we compared functional strength performance across children from three studies that included children with Developmental Coordination Disorder (DCD), Intellectual Disabilities (ID), and Cerebral Palsy (CP) using the Functional Strength Measurement (FSM). As expected, all three cohorts showed lower levels of muscle endurance and muscle power compared to typically developing (TD) children [8,13,14,15,16,25,27,28]. It is noteworthy that these lower scores were observed despite adaptations that were intended to facilitate task comprehension or feasibility in the ID and CP cohorts. Although impairments were found across both muscle-power and muscle-endurance tasks, the pattern of performance differed between clinical cohorts. Exploratory comparisons among the three cohorts indicated significant differences in overarm throwing, underarm throwing, lifting a box and stair climbing, whereas no significant differences were found for the remaining FSM items. The hypothesis that children in the CP cohort would demonstrate the lowest levels of functional strength was partially supported. Significant differences favoring the DCD or ID cohorts were observed for some FSM tasks, but not across all outcomes. These exploratory findings highlight the need to address specific deficits in functional tasks, which could inform targeted intervention strategies. Based on the existing literature, several diagnosis-related constraints have been identified. When this information is considered in relation to the individual items of the FSM, it may provide a hypothetical explanation for why certain items are more challenging for children in each clinical cohort. Further research is needed to substantiate these hypotheses.

### 4.2. Muscle Endurance

All three clinical cohorts performed poorly on the muscle endurance tasks (lateral step-up, sit-to-stand, lifting a box, and stair climbing) compared to the reference norms. When comparing the three clinical cohorts, significant between-cohort differences with medium effect sizes were observed for lifting a box (ID < DCD) and stair climbing (CP < DCD). No significant between-cohort differences were observed for the lateral step-up and sit-to-stand. The endurance tasks in the FSM require both muscle strength and rapid alternation between agonist and antagonist contractions, which can be particularly challenging for children with DCD, ID, and CP [15,17].

Children with DCD struggle with fast, alternating movements due to coordination difficulties, particularly in tasks that require switching between muscle groups [17]. In children with CP, reciprocal muscle contractions are impaired due to alterations in inhibitory pathways, making tasks like stair climbing particularly challenging [15]. In children with Down Syndrome, hypotonia and ligamentous laxity are commonly reported and may contribute to difficulties with rapid alternating movements, compounded by cognitive challenges in maintaining task demands (e.g., speed and duration) [29,30]. Similar motor constraints have been reported in some children with intellectual disabilities, although the underlying mechanisms are likely heterogeneous and may vary depending on the specific diagnosis [29,30]. Poor core muscle strength can make lifting a heavy box more difficult. Children with intellectual disabilities (ID) are known to have lower levels of core strength [27].

Sit-to-stand performance, which predominantly requires muscle endurance, illustrates these challenges. In children with CP, the rising phase is influenced by muscle weakness and altered postural control, while children with DCD and ID also struggle due to muscle weakness. Furthermore, children with CP cannot selectively contract the agonist and relax the antagonist [15]. Dynamic postural control is critical in this task, making it particularly challenging for the DCD, ID, and CP cohorts [23,31,32,33]. Children with CP often compensate with trunk flexion, which reduces efficiency and increases movement duration [32].

### 4.3. Muscle Power

Similar to muscle endurance, children in the DCD, ID, and CP cohorts showed lower levels of muscle power in tasks such as the standing long jump, overarm throwing, and underarm throwing compared to the norms. Comparing the data from the three clinical cohorts, significant between-cohort differences with medium effect sizes were observed for overarm throwing (CP < ID) underarm throwing (CP < ID). No significant between-cohort differences were observed for standing long jump. The reduced throwing performance was observed in children from the CP cohort. The lower performance observed in the CP cohort may be related to neuromuscular impairments commonly reported in children with CP. Reduced muscle strength, impaired antagonist muscle relaxation, and alterations in muscle tissue may limit force generation and efficient force transfer during throwing [15,34]. In addition, restricted joint mobility, particularly at the elbow, may further constrain the throwing motion. Previous research has shown that children with CP demonstrate longer movement durations, reduced elbow range of motion, greater shoulder abduction and flexion, and lower maximal trunk flexion velocity during throwing [35]. These altered movement patterns may indicate compensatory strategies to overcome limitations in strength, mobility, and motor coordination, potentially contributing to reduced throwing distance.

Standing long jump was not significantly different between the three clinical cohorts. Task analysis reveals that performance deficits are due to a combination of factors beyond muscle strength alone. In CP, limitations such as reduced ankle mobility and muscle weakness, particularly in the plantar flexors, hinder explosive power generation. Reduced ankle mobility also affects optimal jumping posture and the ability to propel the body effectively. Additionally, the lack of rapid, symmetric arm swings further limits performance [15,31].

In children with DCD, coordination deficits play a significant role, even when muscle strength is relatively intact [12,36]. Children with ID frequently present with motor coordination deficits, lower-limb power impairments, and underlying neurological features such as hypotonia that markedly impair ballistic movements like the standing long jump [29,30].

### 4.4. Age-by-Cohort Analyses

The second aim of the study was to examine whether the associations between age and functional strength performance differed across the three clinical cohorts (DCD, ID, CP). A significant interaction between cohort and age was found for all explosive power items (overarm throwing, standing long jump and underarm throwing) and for sit-to-stand. These findings indicate that the relation between age and FSM performance was not identical across the three cohorts. For the children in the CP cohort a negative unstandardized regression coefficients was found on standing long jump and sit-to-stand indicating that higher age was not consistently associated with better scores. The positive age-related associations observed in the ID cohort suggest that the relationship between age and performance may differ from that observed in the CP cohort. Longitudinal studies are required to determine whether these cross-sectional associations reflect developmental processes. Previous research has shown that muscle strength development in individuals with cerebral palsy does not keep pace with increases in body mass during growth [37].

In previous research, children with ID reportedly have motor delays due to hypotonia, hyperlaxity, and cognitive impairment [29,30]. It could be that in children with ID the development of an appropriate coordinative pattern to master the task takes longer and the notion of time constraint of the task (as often as possible in 30 s) might also be difficult, which was less of a problem in the older children with ID. However, because the data are cross-sectional it is not possible to determine whether these associations reflect developmental changes. Future longitudinal studies are needed to investigate this.

Positive age-related associations were also observed in the DCD cohort. When examining the regression lines corresponding to the estimated slopes, the lines for the DCD and ID cohorts appear to converge with increasing age. Previous studies have reported larger differences from typically developing peers among older children with DCD. In the present cross-sectional sample, age-related associations were generally positive but varied across tasks [17,24]. Explanations in the literature assume that due to lower participation in physical activities children with DCD have fewer opportunities to develop adequate levels of functional strength. Physical and social barriers may further reinforce negative attitudes and motivational patterns [38].

Given the cross-sectional nature of the data, no conclusions can be drawn about changes in performance over time. Moreover, no correction for multiple testing was applied across the eight age-by-cohort interaction models. These exploratory findings should therefore be interpreted cautiously and require confirmation in independent or longitudinal studies.

### 4.5. Clinical Implications

For clinicians, the findings highlight that children with DCD, ID, and CP all experience limitations in functional-strength activities, although the pattern of limitations appeared to differ across cohorts. The present exploratory results, together with the previous literature, suggest that different functional constraints may influence performance in different populations. Tailoring task-oriented interventions to address the specific limitations in mobility, strength, and coordination is essential. The previous literature suggests that intervention approaches targeting ankle mobility and strengthening plantar flexors in children with CP in a task-oriented way may improve jumping performance, while training rapid alternating movements during skill training in children with DCD can help address endurance limitations.

Given the increased risk of physical inactivity [39,40,41,42], especially in older children with neurodevelopmental disabilities, promoting inclusive or adapted sports activities is critical to maintaining and improving functional strength and overall physical fitness. Early interventions can help prevent the downward spiral of reduced activity and functional decline.

### 4.6. Limitations

In this study we combined data from three previous studies. Although comparable procedures, protocols, and assessment methods were used, study-related influences cannot be completely excluded. In FSM-CP and FSM-ID some small adaptations were made. Therefore, the tasks were slightly different. For example, in lifting a box, children with CP were allowed to support the box with their arm instead of grasping. This adaptation was made due to feasibility of the task and aligns with the strategy children use in daily life. Although the adaptations were intended to preserve the construct being assessed, formal measurement equivalence between FSM, FSM-ID and FSM-CP has not yet been established. Consequently, direct comparisons between clinical cohorts should be interpreted with some caution and considered exploratory in nature. Sample sizes within the individual clinical cohorts were modest, and clinical heterogeneity existed within both the CP and ID cohorts. Furthermore, information regarding physical activity participation, therapy exposure, medication use, and other potentially relevant confounding factors was not available in the original datasets. These factors may have influenced functional strength performance and should be considered in future research. Finally, the cross-sectional nature of the study prevents conclusions regarding developmental trajectories or changes in functional strength over time. Longitudinal studies are needed to clarify the age-related associations observed in the present sample.

### 4.7. Future Research Directions

Future studies should explore the diagnosis-related constraints (e.g., muscle tone, range of motion, postural control) and the level of physical activity that influence performance on functional strength tasks. Future longitudinal studies are required to determine whether the observed age-related associations reflect true developmental trajectories. Additionally, investigating the role of physical activity in maintaining or improving functional strength will help identify effective intervention strategies.

## 5. Conclusions

Children in the DCD, ID, and CP cohorts demonstrated lower levels of functional strength than typically developing peers. Comparing the data of the three cohorts a significant differences were observed for selected FSM tasks, particularly overarm throwing, underarm throwing, lifting a box, and stair climbing. However, as the data were derived from three distinct cohorts rather than collected within a single study, and minor differences exist between FSM, FSM-ID and FSM-CP, the findings should be interpreted with caution and considered hypothesis-generating rather than confirmatory. Age-related associations differed across the clinical cohorts for several functional strength outcomes, indicating that the relationship between age and performance may not be identical across these populations. However, because the data were cross-sectional, these findings should not be interpreted as evidence of developmental change or decline over time. Longitudinal studies are required to further investigate the age-related associations observed in the present sample.

## Figures and Tables

**Table 1:** Demographic information of the three cohorts.

	DCD	ID	CP	*p*-Value
Number (boys/girls)	29 (23/6)	47 (37/10)	27 (16/11)	0.14
Median age (min-max), years	8 (7–10)	9 (7–10)	8 (7–10)	0.47
Median height (min-max), cm	137 (124–152)	136 (106–161)	134 (117–151)	0.63
Median weight (min-max), kg	35 (22–48)	32 (12–65)	27 (20–60)	0.02

DCD: Developmental Coordination Disorder; ID: Intellectual Disabilities; CP: Cerebral Palsy; cm = centimeters; kg = kilogram; SD = standard deviation.

**Table 2:** Description of the Functional Strength measurement (FSM) items and adaptations for different groups.

Item	FSM-DCD	FSM-ID	FSM-CP
**Overarm throwing**	Throwing a heavy bag (2 or 3 kg) as far as possible. Measure the distance in centimeters.	**Adaptation**: Visual cue of a thick line with 2 footsteps behind the line.	**Adaptation**: The affected hand may be used to support the bag instead of grasping it.
**Standing long jump**	Jumping forwards as far as possible. Measure the distance in centimeters.	**Adaptation**: Visual cue of a thick line with 2 footsteps behind the line.	**No adaptions made.**
**Underarm throwing**	Throwing a heavy bag (2 or 3 kg) as far as possible. Measure the distance in centimeters.	**Adaptation**: Visual cue of a thick line with 2 footsteps behind the line.	**Adaptation**: The affected had may be used to support the bag instead of grasping it.
**Lateral step-up**	Touch the foot to the floor as fast as possible. Count the number of repetitions in 30 s. 2 fingers against the wall for keeping balance.	**Adaptation**: Tester sits on the stairs to prevent children from walking over the stairs.	**Adaptation**: More than 2 fingers may be used for support, without leaning against the wall.
**Chest pass**	Pushing a heavy bag (1 or 2 kg) as far as possible. Measure the distance in centimeters.	**No adaptions made.**	**Deleted.**
**Sit-to-stand**	Stand up and sit down as quickly as possible. Count the number of repetitions in 30 s.	**Adaption**: Encourage to stand up and reach the experimenter’s hand.	**No adaptions made.**
**Lifting a box**	Lift a plastic box filled with heavy bags (3 or 4 kg) onto a wooden box as quickly as possible. Count the number of repetitions in 30 s.	**No adaptions made.**	**Adaptation**: The affected had may be used to support the plastic box instead of grasping it.
**Stair climbing**	Climbing up and down the stairs as quickly as possible. Count the number of steps in 30 s.	**No adaptions made.**	**No adaptations made.**
Test-retest reliability	ICC 0.91–0.94	ICC 0.89–0.94	ICC 0.79–0.95
Construct validity	FSM-HHD r = 0.42–0.74. FSM-MABC-2 r = 0.23–0.39	FSM-HHD r = 0.039–0.79 FSM-BOT-2 strength r = 0.041–0.80 FSM-BOT2 balance r = 0.041–0.71	FSM-HHD r = 0.36–0.69 GMFCS1 > GMFCS2

BOT-2 = Bruininks-Oseretsky Test of motor proficiency, second edition; CP = Cerebral Palsy; ICC = interclass correlation coefficient; GMFCS = gross motor function classification system; HHD = held dynamometer; ID = Intellectual Disabilities; r = correlation coefficient.

**Table 3:** Differences between clinical cohorts on the items of the Functional Strength Measurement.

Outcome	DCD (*n* = 28) Mean (SD)	ID (*n* = 38) Mean (SD)	CP (*n* = 25) Mean (SD)	*F*(2, 87)	*p*	ηp^2^	Significant Pairwise Comparison †
Overarm throwing (cm)	213 (46)	219 (67)	169 (36)	3.95	0.023	0.083	CP < ID
Standing long jump (cm)	98 (27)	91 (25)	82 (28)	2.58	0.082	0.056	—
Underarm throwing (cm)	277 (68)	289 (88)	223 (63)	3.28	0.042	0.070	CP < ID
Lateral step-up best (*n*/30 s)	29 (7)	28 (7)	27 (7)	0.16	0.850	0.004	—
Lateral step-up other (*n*/30 s)	25 (7)	26 (7)	24 (6)	0.52	0.598	0.012	—
Sit-to-stand (*n*/30 s)	19 (4)	20 (4)	20 (5)	0.06	0.939	0.001	—
Lifting a box (*n*/30 s)	20 (5)	17 (6)	18 (6)	4.44	0.015	0.093	ID < DCD
Stair climbing (steps/30 s)	60 (11)	54 (13)	48 (15)	4.85	0.010	0.100	CP < DCD

CP = cerebral palsy; ID = intellectual disability; DCD = developmental coordination disorder; cm = centimeter; *n*/30 s = number of repetitions in 30 s; step/30 s = number of steps in 30 s; ηp^2^ = Partial η^2^ represents the effect size for the omnibus group effect. † Pairwise comparisons are Bonferroni-adjusted (*p* < 0.05). Significant comparisons were CP versus ID for overarm throwing (*p* = 0.034) and underarm throwing (*p* = 0.047), ID versus DCD for lifting a box (*p* = 0.017), and CP versus DCD for stair climbing (*p* = 0.008).

**Table 4:** Interaction of age, clinical cohorts and age-by-cohort on the different FSM-items Sample sizes vary across outcomes because participants unable to complete a specific task were excluded only from that analysis.

Test Item	Age *F*	*p*	ηp^2^	Cohort *F*	*p*	ηp^2^	Cohort × Age *F*	*p*	ηp^2^	DCD *B*	ID, *B*	CP, *B*
Overarm throwing (*n* = 103)	22.06	<0.001	0.185	2.88	0.060	0.056	3.18	0.046	0.062	1.481	2.732	0.782
Standing long jump (*n* = 101)	1.44	0.233	0.015	3.65	0.030	0.071	3.91	0.023	0.076	0.449	0.760	−0.494
Underarm throwing (*n* = 102)	17.96	<0.001	0.158	3.39	0.038	0.066	3.84	0.025	0.074	1.917	3.858	0.744
Lateral step-up best (*n* = 98)	4.64	0.034	0.048	2.66	0.075	0.055	2.67	0.075	0.055	0.181	0.215	−0.055
Lateral step-up other (*n* = 97)	2.24	0.138	0.024	2.83	0.064	0.059	3.09	0.050	0.064	0.133	0.205	−0.100
Sit-to-stand (*n* = 97)	0.21	0.652	0.002	6.01	0.004	0.117	5.83	0.004	0.114	0.075	0.107	−0.139
Lifting a box (*n* = 103)	14.22	<0.001	0.128	1.14	0.325	0.023	0.43	0.655	0.009	0.115	0.193	0.135
Stair climbing (*n* = 102)	4.35	0.040	0.043	2.52	0.086	0.050	2.82	0.065	0.055	0.274	0.425	−0.095

CP = cerebral palsy; ID = intellectual disability; DCD = developmental coordination disorder; B = regression coefficients represent cohort-specific estimated regression slopes per month of age, expressed in the units of the respective FSM items; ηp^2^ = partial eta square.; ηp^2^ = partial eta square.

## Data Availability

The datasets used and/or analysed during the current study are available from the corresponding author on reasonable request due to ethical considerations. The important data supporting the conclusions of this article are included within the article (Table 3 and Table 4).

## Abbreviations

The following abbreviations are used in this manuscript:

TD	Typical Developing
CP	Cerebral Palsy
DCD	Developmental Coordination Disorder
ICF	International Classification of Functioning, Disability and Health
